# Associations between neutering and early‐onset urinary incontinence in UK bitches under primary veterinary care

**DOI:** 10.1111/jsap.13072

**Published:** 2019-10-07

**Authors:** C. Pegram, D. C. Brodbelt, D. B. Church, J. Hall, L. Owen, Y.‐M. Chang, D. G. O'Neill

**Affiliations:** ^1^ Pathobiology and Population Science The Royal Veterinary College Hatfield Herts AL9 7TA UK; ^2^ Clinical Sciences and Services The Royal Veterinary College Hatfield Herts AL9 7TA UK; ^3^ Royal (Dick) School of Veterinary Studies University of Edinburgh, Easter Bush Roslin EH25 9RG UK; ^4^ Department of Veterinary Medicine University of Cambridge Cambridge CB3 0ES UK; ^5^ Research Support Office The Royal Veterinary College Hatfield Herts AL9 7TA UK

## Abstract

**Objectives:**

To investigate association between neutering and early‐onset urinary incontinence in bitches under primary veterinary care in the UK.

**Materials and Methods:**

A retrospective cohort study of bitches within VetCompass born between January 1, 2010 and December 31, 2012 that were followed until March 31, 2018. The clinical records were automatically searched and manually validated for incontinence cases. Incidence risk and rate over the study period were calculated. Cox regression modelling separately evaluated the hazard of urinary incontinence and association with neutering: (1) from the date of birth for all bitches, both neutered and entire; and, (2) from the date of neutering for the neutered subset. Other variables considered included breed, bodyweight and veterinary practice group.

**Results:**

Overall, 492 bitches were identified with early‐onset urinary incontinence from a total of 72,971 included in the study period. Incidence risk was 0.68% (95% confidence intervals 0.62 to 0.74), while incidence rate increased with age. After accounting for confounding factors, increased hazard of early‐onset urinary incontinence was identified in: (1) neutered bitches, with the effect increasing with age; and, (2) bitches neutered before 6 months, within the first 2 years following neutering. In both models, increased hazard was additionally associated with increasing bodyweight and breed.

**Clinical Significance:**

Neutering itself and early‐age neutering (<6 months) are major risk factors for early‐onset urinary incontinence. These results should be taken into account in making evidence‐based recommendations on neutering and its timing.

## INTRODUCTION

Urinary incontinence (UI) is defined as the involuntary leakage of urine during the storage phase of micturition (Schaer 2010). Bitches usually present either as juveniles with congenital UI or, more commonly, as adults with acquired UI (Holt 1990). Juvenile cases have been described as puppies with UI noted from birth up to 6 months old, with adult cases described as continent during puppyhood but developing UI later in life (Holt 1985). In the juvenile bitch, UI results most commonly from ectopic ureters while urethral sphincter mechanism incompetence (USMI) is the most common cause in the adult (Holt & Thrusfield 1993, Gregory 1994).

A complete history and diagnostic work up, including urinalysis, urine culture and imaging studies, is ideally required to confirm the underlying cause of UI (Silverman & Long 2000). However, in adult bitches a presumptive diagnosis of USMI as a cause of UI is often made based on signalment, history, physical examination, urinalysis (± urine culture) and response to USMI‐specific treatment (Gregory 1994, Silverman & Long 2000). Instead of accepting a presumptive diagnosis of USMI, referring to UI overall rather than to precise subsets avoids assumptions regarding causality.

The prevalence (all cases) and incidence (new cases) of UI in bitches have been reported at differing frequencies, ranging from 3.14 to 20.1% (Arnold 1997, O'Neill *et al*. 2017). However, these reported estimates are difficult to compare directly because of the widely differing study designs, populations, settings, periods and case definitions used across publications. UI prevalence in bitches (both neutered and entire) was estimated to be 3.14% in primary‐care practice in England (O'Neill *et al*. 2017). This estimate was based on a cross‐sectional study design that was the largest primary‐care study of bitches (n=100,397) to date and therefore may offer reliable prevalence data and also risk factor analysis for time‐invariant factors such as breed. However, cohort study design is needed to better assess associations for risk factors that change over time, such as neuter status (Dohoo *et al*. 2009).

USMI, the major cause of acquired UI in adult bitches, has a complex and poorly understood pathophysiology, with anatomical, hormonal and neurological abnormalities all suggested to play developmental roles (Gregory 1994, Coit *et al*. 2008, de Bleser *et al*. 2011). Although neutering has been widely cited as a risk factor for USMI (Holt 1985, Holt & Thrusfield 1993, Thrusfield *et al*. 1998, O'Neill *et al*. 2017), the earlier evidence supporting this association was reported as weak in a systematic review (Beauvais *et al*. 2012). The median/mean time from neuter to onset of UI has been reported as ranging from 2.3 years to 5.0 years (de Bleser *et al*. 2011, Forsee *et al*. 2013).

Although pathophysiological mechanisms that might support association between neutering and acquired UI are not fully elucidated, there is evidence of a relationship between gonadotrophin concentrations and continence (Reichler *et al*. 2005). Gonadotrophins, along with oestrogen, may be involved in regulating bladder tone and maintaining urethral wall thickness, thus creating a more efficient urethral seal (Ponglowhapan *et al*. 2007). Reduction in expression of gonadotrophin receptors, specifically luteinising hormone and follicle‐stimulating hormone, as well as cyclooxygenase‐2 receptors has been reported in neutered bitches. It has been suggested that lower receptor expression may have negative effects on the physiological function of the bladder and urethra (Ponglowhapan *et al*. 2007). In further reports, neutered bitches had increased collagen and reduced glycosaminoglycan (GAG) components in their lower urinary tract (LUT) compared to intact bitches and these changes may compromise structural and functional integrity of the LUT in neutered bitches and may promote UI after neutering (Ponglowhapan *et al*. 2008, 2011).

Associations between the age at neuter and subsequent UI in the bitch have been investigated. Increasing age at neuter was associated with reducing risk of UI up until 12 months of age in one study, after which there was no further effect (Spain *et al*. 2004), but the quality of this evidence was considered weak in a systematic review (Beauvais *et al*. 2012). A recent US study reported a more nuanced relationship between the age at neutering and the risk of UI. Bitches weighing >25 kg that were neutered within their first year had significantly decreased hazard of UI for every 1‐month delay in neutering. For a 25 kg bitch, a 1‐month delay in neuter age decreased the hazard of incontinence by 11%. However, for bitches <15 kg, the hazard of UI was not associated with the age at neuter (Byron *et al*. 2017). In a recent study focusing solely on German shepherd dogs (172 entire females, 293 neutered females), the incidence of UI was 7% in females neutered before 1 year. In females neutered at 1 year and beyond, the incidence of UI dropped, and was not diagnosed at all in intact females (Hart *et al*. 2016).

Breed, bodyweight and age have been reported as risk factors for UI. A recent UK study reported the highest odds in the Irish setter, Dobermann, bull mastiff, rough collie, Dalmatian and boxer (O'Neill *et al*. 2017). Earlier reports additionally reported the old English sheepdog, Rottweiler and Weimaraner at high risk (Holt & Thrusfield 1993). Increased bodyweight is associated with increased risk of UI (de Bleser *et al*. 2011, O'Neill *et al*. 2017), with heavier dogs (>15 kg) having approximately seven times the risk of acquired UI compared with lighter dogs (<15 kg) in one study (Forsee *et al*. 2013). Increasing age has been associated with an increased risk of UI in several studies (Thrusfield *et al*. 1998, Stocklin‐Gautschi *et al*. 2001, de Bleser *et al*. 2011, O'Neill *et al*. 2017). The structural and functional ability of the urinary system deteriorates as animals age, which can contribute to UI in the geriatric bitch (Krawiec 1989).

Predisposition to UI has been reported as a belief by veterinarians as a major contra‐indication of neutering bitches, second only to obesity (Diesel *et al*. 2010). However, this perception is not universally accepted across the veterinary profession and clients often receive conflicting veterinary advice on the optimal neutering practices, with mixed views on whether bitches should be neutered at all and, if so, at what age (Diesel *et al*. 2010). Using veterinary clinical data from the VetCompass Programme (VetCompass 2018), this retrospective cohort study aimed to explore association between neuter status and age at neuter with early‐onset UI, after taking other demographic risk factors into account. This would provide relevant and reliable evidence that can support a more universal approach from veterinary surgeons and providing advice to dog owners regarding neutering.

## METHODS

The VetCompass Programme collects anonymised electronic patient record (EPR) data from primary‐care veterinary practices in the UK for epidemiological research (VetCompass 2018). Information available for VetCompass researchers includes a unique ID for each animal with concomitant species, breed, date of birth, sex and neuter status. Clinical information from free‐text clinical notes, summary diagnosis terms (VeNom codes (The VeNom Coding Group 2019)), bodyweight data and treatment with corresponding dates are also available.

A retrospective cohort study was designed to evaluate factors associated with time to development of early‐onset UI. Inclusion in the cohort study required that the dog was female, born from January 1, 2010 to December 31, 2012 inclusive and had at least one EPR before 12 months of age available in the VetCompass database. Bitches were followed over time in the clinical records for up to 8 years, with the end of the study period on March 31, 2018. Sample size calculations *in Epi info (CDC)* estimated that approximately 3328 bitches would be needed to detect a significant risk ratio of ≥2, based on an estimated 1.5% of entire animals developing UI during the study period, assuming 80% power and 95% confidence with a 1:1 ratio of neutered to entire bitches. A 1:1 ratio was chosen based on primary‐care demographic data reported in a VetCompass study (O'Neill *et al*. 2014). Ethics approval was obtained from the RVC Ethics and Welfare Committee (reference number 2015/1369).

Inclusion as a UI case required a final diagnosis of UI (or synonym) recorded in the EPR and/or treatment with either phenylpropanolamine or oestriol. Exclusion criteria comprised: UI recorded as occurring secondary to a primary neurological condition, evidence of urinary tract infection with UI reported to resolve with appropriate treatment of the urinary tract infection, or evidence that the phenylpropanolamine or oestriol was given for a reason other than UI. Early‐onset UI was defined as UI (according to the inclusion and exclusion criteria) diagnosed at ≤8 years.

Candidate UI cases were identified using search terms appropriate to the diagnosis and management of UI in the clinical notes (incont*, usmi, urin* leak*, incompet*, nocturia, urethral sp*, wetting, wet* bed, drib* urin*, inapprop* urin*) and brand names in the treatment fields (propal*, incuri*, urilin, enurace). The search findings were merged and the clinical notes of all candidate cases were examined manually in detail to identify all confirmed UI cases that met the case definition. Candidate cases that did not meet the UI definition and the remaining non‐candidate bitches were classified as non‐cases and were included in the study as the comparator group. Demographic and treatment data for cases and non‐cases were extracted automatically from the VetCompass database. Additional data were also extracted where available from the EPRs, including the date of any neuter surgery for cases and non‐cases and the date of first diagnosis of UI for cases. Bitches that were not diagnosed with UI by the end of the study period were censored on the date of their final EPR.

Bitches were categorised into a “Breed” variable using standardised breed terms (The VeNom Coding Group 2019). “Designer breeds” described contrived breed names that were contractions of two or more other standardised purebred breed names, for example, Labradoodle as a contraction of Labrador retriever and poodle (Oliver & Gould 2012). In order to maintain sufficient power for breed‐based analyses, the breed variable included specific breeds with five or more UI cases and/or breeds with ≥1500 animals overall. Remaining bitches were grouped in to “Purebred – Other”, “Crossbred – designer” or “Crossbred – non‐designer”. Neuter status was examined as “Neutered” and “Entire”, with the status taken for animals with available data at the date of UI diagnosis for cases and final EPR for non‐cases. Therefore, any case neutered after first presenting with UI was considered entire in the analysis. Bodyweight (kg) described the recorded value closest to the date of diagnosis for cases and closest to the date of final EPR for non‐cases. Based on previous literature in the subject area (O'Neill *et al*. 2017, Pegram *et al*. 2019), bodyweight (kg) was categorised into five groups: < 10, 10 to <20, 20 to <30 and ≥ 30 kg, with missing values grouped as “Not recorded”. Non‐adult bodyweights were included in the study, because risk factors at the point of UI diagnosis were of primary interest. Veterinary group attended was categorised as 1 to 3, based on the three practice groups involved in the study. Based on previous literature in the subject area (Pegram *et al*. 2019), age at neuter (months) was calculated at the date of neuter surgery and categorised into four groups: < 6, 6 to <12, 12 to <24 and ≥24. Data were checked and cleaned in Excel (Microsoft Corp), before export to SPSS version 24.0 (IBM Corp) for statistical analysis.

Descriptive statistics were generated for UI cases and non‐cases. Incidence risk for the study period and incidence rate within different age categories (0 to <2 years, 2 to <4 years, 4 to <6 years and 6 to 8 years) with corresponding 95% confidence intervals (95% CIs) were calculated. Normality was assessed graphically and continuous variables were summarised using median, interquartile range (IQR) and range. Mann‐Whitney U test and Fisher's exact test were used as appropriate for comparison of demographic data between cases and non‐cases (Fisher 1922, Mann & Whitney 1947). Kaplan–Meier survival (*i.e*. time‐to‐event) curves were separately constructed to describe: (1) time to UI from birth; and, (2) time to UI from neutering, and log‐rank tests were used for comparisons between categories (Kaplan & Meier 1958).

Cox regression modelling was used to evaluate the hazard of diagnosis with UI for association with the two risk factors of primary interest (neuter status and age at neuter) in two separate models (Cox 1972). Cox regression modelling for “neuter status” as a binary risk factor used the entire dataset, with the time start point set as date of birth. Cox regression modelling for “age at neuter” as a categorical risk factor used just the neutered subset of bitches (because this risk factor could not apply to entire bitches), with the time start point set as date of neuter. Other variables considered in the modelling included breed, bodyweight and vet group. Explanatory variables with liberal univariable association with a diagnosis of UI (P<0.2) were carried forward for multivariable Cox regression modelling. Multivariable model building used a manual backwards stepwise approach. First order pairwise interactions between final model explanatory variables were evaluated. The proportional hazards assumption (*i.e*. the assumption that the hazard ratios (HRs) for category variables remain constant over time) was tested by creating time‐by‐covariate interactions for each variable in the model and through visual inspection of log‐cumulative hazard and Kaplan–Meier Cox plots. An interaction with time (in years) was added to the model when a variable violated the proportional hazards assumption (Dohoo 2010). Model fit was assessed using DfBetas to estimate the change in a coefficient if a case was removed. HR and its 95% CI were reported. Final statistical significance was set at the 5% level.

## RESULTS

The study included 77,138 bitches born January 1, 2010 to December 31, 2012 inclusive. Of these, 27,666 (35.9%) were neutered and 49,472 (64.1%) were entire at the end of the study period. Of the 27,666 neutered bitches, 4167 (15.1%) did not have a date of neutering surgery recorded in the EPR and were excluded from further analysis, leaving 23,499 (32.2%) neutered and 49,472 (67.8%) entire (total 72,971 bitches) in the analysis. These 72,971 bitches were followed over time from their first available clinical record before 12 months of age and 493 (0.68%) were identified that met the UI case definition before the end of the study period. Data completeness for the 77,138 bitches were: breed 99.9%, age 100.0%, neuter status 100.0% and bodyweight 83.1%.

Descriptive results were reported on 493 UI cases and 72,478 non‐cases. Incidence risk during the overall study period was 0.68% (95% CI 0.62 to 0.74). Incidence rate increased with each category of increasing age. The incidence rate was 0.0018 per animal‐year for dogs aged 0 to <2 years (95% CI 0.0015 to 0.0021), 0.0030 for dogs aged 2 to <4 years (95% CI 0.0026 to 0.0035), 0.0035 for dogs aged 4 to <6 years (95% CI 0.0029 to 0.0041) and 0.0054 for dogs aged 6 to 8 years (95% CI 0.0039 to 0.0073). The median age at first diagnosis of early‐onset UI was 2.9 years (IQR 1.6 to 4.5, range 0.1 to 7.6), which was higher than the median age at censoring of non‐cases of 1.5 years (IQR 0.4 to 5.0, range 0.1 to 8.2) (P<0.001). Of the non‐cases, 28,324 (39.1%) had follow‐up time beyond the median age at first diagnosis of early‐onset UI (2.9 years). The median bodyweight of cases at first diagnosis of early‐onset UI was 21.3 kg (IQR 12.5 to 30.0, range 1.2 to 70.9), which was higher than the median bodyweight at censoring of 8.5 kg of non‐cases (IQR 4.7 to 17.8, range 0.1 to 91.0) (P<0.001). A higher proportion of cases, 83.6% (412) were neutered compared with 31.9% (23,087) of non‐cases (P<0.001). The most common breeds amongst cases were the Labrador retriever (12.2%; 60), German shepherd dog (4.5%; 22) and Staffordshire bull terrier (3.9%; 19) in addition to 95 (19.3%) non‐designer crossbreds. The most common breeds amongst non‐cases were the Staffordshire bull terrier (9.6%; 6975), Labrador retriever (6.1%; 4426), Jack Russell terrier (5.7%; 4119) and shih‐tzu (4.2%; 3080) in addition to 15,446 (21.3%) non‐designer crossbreds. Of the neutered animals, the median age at neuter was 8.9 months (IQR 6.3 to 14.9, range 4.1 to 69.5) for cases which was younger than for non‐cases (10.3 months, IQR 6.4 to 17.4, range 2.1 to 93.3) (P=0.010). The median time between neuter and first diagnosis of early‐onset UI was 1.9 years (IQR 1.0 to 3.4, range 0 to 7.13).

### Survival (time‐to‐event) and hazard for UI diagnosis in neutered and entire bitches from the date of birth (model 1)

**Figure 1:**
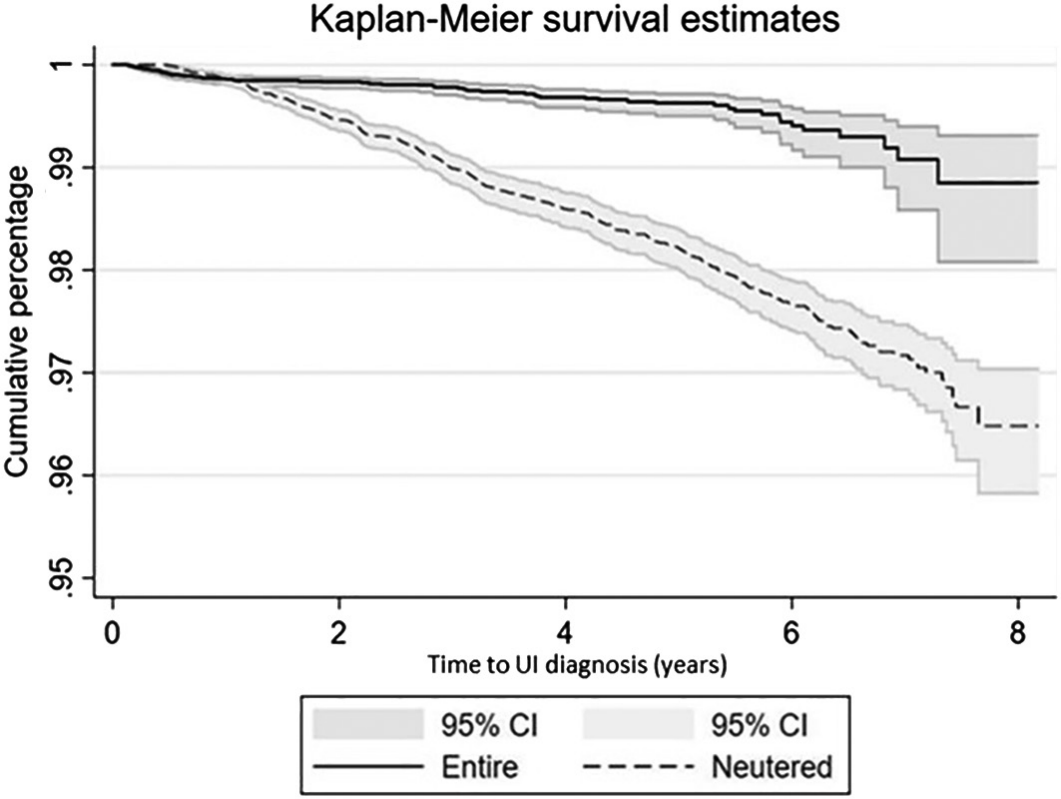
Kaplan–Meier survival curve of early‐onset urinary incontinence diagnosis in neutered and entire bitches attending primary‐care practices in the UK. Survival time represents the time from date of birth until first diagnosis of early‐onset urinary incontinence or censoring

Comparing time‐to‐event from date of birth to early‐onset UI diagnosis, there was evidence of a shorter time to early‐onset UI diagnosis in neutered cases compared with entire cases (log‐rank test, P<0.001) (Fig 1). Univariable Cox regression for the 72,971 bitches born between January 1, 2010 and December 31, 2012 to assess “neuter status” as a risk factor for early‐onset UI diagnosis identified four variables that were carried forward for multivariable modelling: neuter status, bodyweight, breed and vet group (Table 1). The final multivariable model retained all four variables, including a time‐dependent covariate for neuter status, to account for the evidence that the proportional hazards assumption was violated because neuter status had a statistically significant time‐dependent effect (Table 2). No highly influential points that significantly altered coefficient estimates for neuter status were detected. After adjusting for the other demographic risk factors, neutered bitches had 2.12 (95% CI 1.36 to 3.29; P=0.001) times the hazard of early‐onset UI diagnosis from the date of birth compared with entire bitches within the first year of age. For every subsequent year of age, the hazard of UI increased 1.23‐fold (95% CI 1.05 to 1.43; P=0.010) for neutered bitches compared to entire bitches (*e.g*. HR year 2 = 2.12 × 1.23 = 2.61, HR year 3 = 2.12 × 1.23 × 1.23 = 3.21).

**Table 1:** Descriptive statistics and univariable Cox regression results for risk factors associated with the hazard of early‐onset urinary incontinence in bitches under primary veterinary care in the UK (start date: date of birth) (n=72,971)

Variable	Category	Non‐case no.	Case no.	Hazard ratio	95% CI	Category P‐value	Variable P‐value
Neuter status	Entire	49,391	81	Base			<0.001
	Neutered	23,087	412	3.37	2.65 to 4.30	<0.001	
Bodyweight (kg)	<10.0	33,766	92	Base			<0.001
	10.0 to <20.0	13,711	117	2.06	1.57 to 2.71	<0.001	
	20.0 to <30.0	7869	144	3.82	2.94 to 4.97	<0.001	
	≥30.0	4869	120	4.29	3.27 to 5.63	<0.001	
	Not recorded	12,263	20	2.22	1.36 to 3.62	0.001	
Breed	Crossbreed – non‐designer	15,446	95	Base			<0.001
	Irish setter	43	6	19.07	8.36 to 43.51	<0.001	
	Dalmatian	261	11	6.18	3.31 to 11.54	<0.001	
	Hungarian vizsla	146	6	5.48	2.40 to 12.51	<0.001	
	Dobermann	228	6	4.48	1.96 to 10.23	<0.001	
	Weimaraner	204	7	4.38	2.03 to 9.44	<0.001	
	Shar‐pei	468	6	3.09	1.36 to 7.06	0.007	
	Boxer	893	17	2.90	1.73 to 4.87	<0.001	
	English springer spaniel	1503	28	2.55	1.67 to 3.88	<0.001	
	Rottweiler	898	10	2.55	1.33 to 4.89	0.005	
	Bulldog	817	8	2.24	1.09 to 4.60	0.029	
	German shepherd dog	2054	22	1.89	1.19 to 3.01	0.007	
	Labrador retriever	4426	60	1.73	1.25 to 2.39	0.001	
	Border collie	1700	18	1.43	0.86 to 2.37	0.164	
	West Highland white terrier	1114	12	1.28	0.70 to 2.34	0.416	
	Golden retriever	602	6	1.18	0.52 to 2.69	0.696	
	Siberian husky	1002	6	1.16	0.51 to 2.65	0.724	
	Beagle	666	5	1.08	0.44 to 2.65	0.867	
	Purebreed – other	16,046	100	0.96	0.73 to 1.27	0.780	
	Crossbreed – designer	1929	15	0.90	0.52 to 1.55	0.698	
	Staffordshire bull terrier	6975	19	0.61	0.37 to 1.00	0.049	
	Cocker spaniel	2471	10	0.49	0.26 to 0.94	0.032	
	Jack Russell terrier	4119	13	0.47	0.26 to 0.84	0.010	
	Chihuahua	2841	3	0.20	0.06 to 0.64	0.007	
	Yorkshire terrier	2495	3	0.18	0.06 to 0.57	0.003	
	Shih‐tzu	3080	1	0.05	0.01 to 0.35	0.003	
	Not recorded	51	0	~	~	~	
Vet group	1			Base			0.003

CI Confidence interval

**Table 2:** Final Cox regression multivariable model for risk factors associated with the hazard of early‐onset urinary incontinence in bitches under primary veterinary care in the UK – including “neuter status” variable (with date of birth as the start point) (n=72,971)

Variable	Category	Hazard ratio	95% CI	Category P‐value	Variable P‐value
Neuter status	Entire	Base			0.001
	Neutered	2.12	1.36 to 3.29	0.001	
Time‐dependent effect Neuter status	Entire X Time interaction	Base			0.010
	Neutered X Time interaction	1.23	1.05 to 1.43	0.010	
Bodyweight (kg)	<10.0	Base			<0.001
	10.0 to <20.0	1.66	1.22 to 2.25	0.001	
	20.0 to <30.0	2.34	1.70 to 3.24	<0.001	
	≥ 30.0	2.62	1.86 to 3.69	<0.001	
	Not recorded	2.83	1.67 to 4.79	<0.001	
Breed	Crossbreed – non‐designer	Base			<0.001
	Irish setter	15.51	6.73 to 35.75	<0.001	
	Dalmatian	3.94	2.08 to 7.45	<0.001	
	Hungarian vizsla	3.64	1.58 to 8.38	0.002	
	Dobermann	3.26	1.40 to 7.57	0.006	
	Shar‐pei	3.20	1.39 to 7.37	0.006	
	Weimaraner	2.66	1.22 to 5.81	0.014	
	English springer spaniel	2.15	1.39 to 3.31	0.001	
	Bulldog	2.02	0.97 to 4.20	0.060	
	Boxer	1.97	1.16 to 3.35	0.012	
	Rottweiler	1.96	0.99 to 3.86	0.053	
	West Highland white terrier	1.67	0.91 to 3.08	0.100	
	German shepherd dog	1.32	0.81 to 2.15	0.272	
	Border collie	1.15	0.69 to 1.92	0.588	
	Purebreed – other	1.11	0.84 to 1.48	0.462	
	Labrador retriever	1.08	0.76 to 1.54	0.650	
	Siberian husky	0.97	0.42 to 2.23	0.941	
	beagle	0.91	0.37 to 2.25	0.839	
	Crossbreed – designer	0.76	0.44 to 1.31	0.318	
	Golden retriever	0.75	0.33 to 1.75	0.508	
	Jack Russell terrier	0.70	0.38 to 1.27	0.239	
	Staffordshire bull terrier	0.57	0.34 to 0.94	0.026	
	Cocker spaniel	0.44	0.23 to 0.86	0.016	
	Chihuahua	0.38	0.12 to 1.23	0.106	
	Yorkshire terrier	0.31	0.10 to 1.01	0.051	
	Shih‐tzu	0.07	0.01 to 0.52	0.009	
Vet group	1	Base			0.037

Neuter status interacted with time

CI Confidence interval

Eight breeds had increased hazard of early‐onset UI compared to non‐designer crossbreds: Irish setter (HR 15.51; 95% CI 6.73 to 35.75; P<0.001), Dalmatian (HR 3.94; 95% CI 2.08 to 7.45; P<0.001), Hungarian vizsla (HR 3.64; 95% CI 1.58 to 8.38; P=0.002), Dobermann (HR 3.26; 95% CI 1.40 to 7.57; P=0.006), shar‐pei (HR 3.20; 95% CI 1.39 to 7.37; P=0.006), Weimaraner (HR 2.66; 95% CI 1.22 to 5.81; P=0.014), English springer spaniel (HR 2.15; 95% CI 1.39 to 3.31; P=0.001) and boxer (HR 1.97; 95% CI 1.16 to 3.35; P=0.012). Reduced hazard was observed for the Staffordshire bull terrier (HR 0.57; 95% CI 0.34 to 0.94; P=0.026), cocker spaniel (HR 0.44; 95% CI 0.23 to 0.86; P=0.016) and shih‐tzu (HR 0.07; 95% CI 0.01 to 0.52; P=0.009). Increasing bodyweight (kg) was associated with an increasing hazard of early‐onset UI diagnosis, with bitches weighing ≥30.0 kg having 2.62 times the hazard (95% CI 1.86 to 3.69; P<0.001) compared with those <10.0 kg. In addition, vet group was identified as a statistically significant variable (P=0.037) and was retained in the multivariable model to account for differences between practice groups (Table 2). As this variable was included as a fixed effect and not as a primary risk factor, the individual category P‐values are not reported.

### Survival (time‐to‐event) and hazard for early‐onset UI diagnosis in the neutered subset of bitches from the date of neutering (model 2)

**Figure 2:**
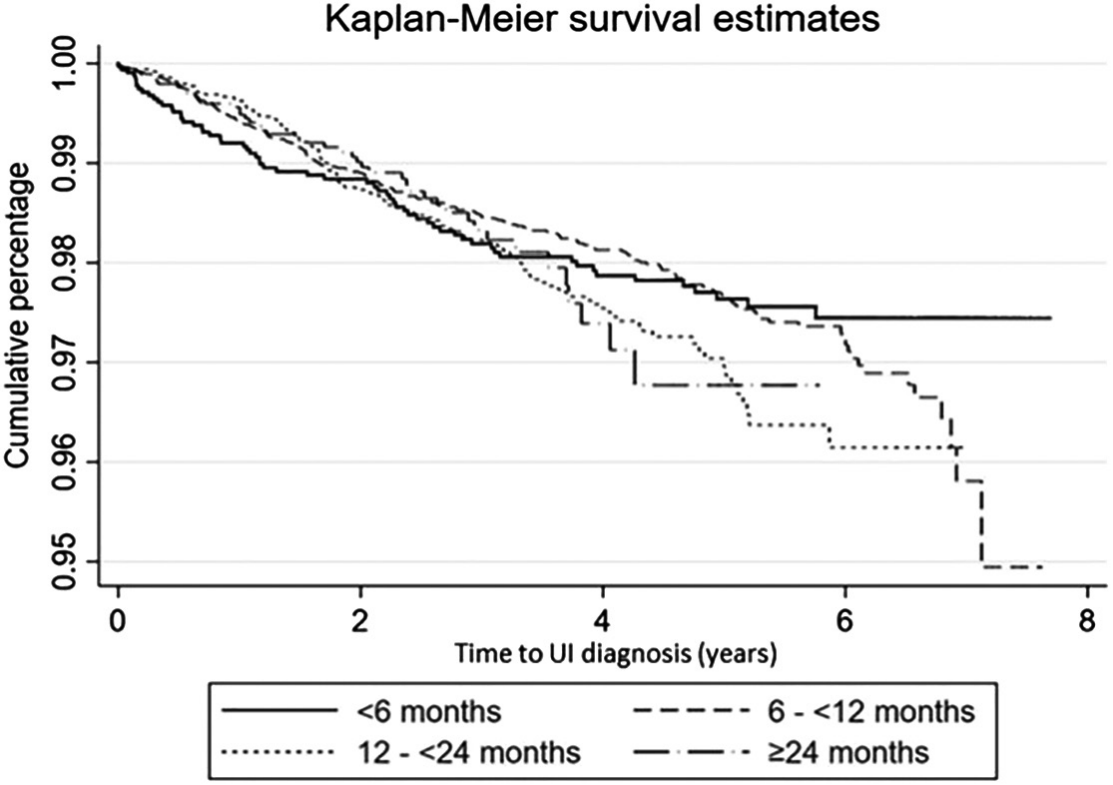
Kaplan–Meier survival curve of early‐onset urinary incontinence diagnosis in bitches neutered at different ages attending primary‐care practices in the UK. Survival time represents the time from date of neuter until first diagnosis of urinary incontinence or censoring

Comparing *time‐to‐event* from date of neuter to the date of early‐onset UI diagnosis, there was limited evidence of differing times to early‐onset UI between the four categories of “age at neuter” in the neutered‐only subset of bitches (log‐rank test, P=0.188) (Fig 2). Univariable Cox regression using the neutered‐only subset to assess “age at neuter” as a risk factor for early‐onset UI diagnosis identified four variables that were carried forward for multivariable modelling: age at neuter, bodyweight, breed and vet group (Table 3). The final multivariable model retained all four variables, including a time‐dependent covariate for age at neuter, to account for the evidence that the proportional hazards assumption was violated because the age at neuter had a statistically significant time‐dependent effect (Table 4). No highly influential points that significantly altered coefficient estimates for age at neuter were detected.

**Table 3:** Descriptive statistics and univariable Cox regression results for risk factors associated with the hazard of early‐onset urinary incontinence in the neutered‐only subset of bitches under primary veterinary care in the UK (with date of neuter as the start point) (n=23,499)

Variable	Category	Non‐case no.	Case no.	Hazard ratio	95% CI*	Category P‐value	Variable P‐value
Age at neuter (months)	<6	3355	63	0.94	0.71 to 1.25	0.665	0.190
	6–<12	10,352	191	Base			
	12–<24	5313	113	1.25	0.99 to 1.58	0.059	
	≥24	4067	45	1.12	0.81 to 1.56	0.492	
Bodyweight (kg)	<10.0	9251	67	Base			<0.001
	10.0 to <20.0	6489	96	1.98	1.45 to 2.71	<0.001	
	20.0 to <30.0	4079	130	4.18	3.12 to 5.62	<0.001	
	≥ 30.0	2692	110	4.84	3.57 to 6.56	<0.001	
	Not recorded	576	9	7.79	3.88 to 15.64	<0.001	
Breed	Crossbreed – non‐designer	5486	77	Base			<0.001
	Irish setter	10	5	26.49	10.72 to 65.49	<0.001	
	Dalmatian	87	11	7.78	4.14 to 14.63	<0.001	
	Hungarian vizsla	50	5	6.07	2.46 to 15.01	<0.001	
	Dobermann	59	5	6.02	2.44 to 14.88	<0.001	
	Weimaraner	74	7	5.56	2.56 to 12.05	<0.001	
	Rottweiler	173	10	5.10	2.64 to 9.85	<0.001	
	Boxer	262	13	3.16	1.76 to 5.69	<0.001	
	English springer spaniel	521	24	2.94	1.86 to 4.65	<0.001	
	German shepherd dog	536	20	2.72	1.66 to 4.44	<0.001	
	Siberian husky	223	6	2.22	0.97 to 5.09	0.061	
	Labrador retriever	1760	51	1.80	1.27 to 2.57	0.001	
	Border collie	648	16	1.57	0.92 to 2.69	0.101	
	West Highland white terrier	466	11	1.40	0.74 to 2.63	0.300	
	Staffordshire bull terrier	1588	19	1.03	0.62 to 1.70	0.919	
	Purebreed – other	7637	97	0.85	0.63 to 1.15	0.283	
	Crossbreed – designer	1015	14	0.83	0.47 to 1.47	0.519	
	Cocker spaniel	1030	10	0.57	0.30 to 1.11	0.099	
	Jack Russell terrier	1459	11	0.52	0.28 to 0.98	0.043	
	Not recorded	3	0	~	~	~	
Vet group	1			Base			0.022

CI Confidence interval

**Table 4:** Final multivariable Cox regression model for risk factors associated with the hazard of early‐onset urinary incontinence in the neutered‐only subset of bitches under primary veterinary care in the UK – including “age at neuter” variable with date of neuter as the start point (n = 23,499)

Variable	Category	Hazard ratio	95% CI	Category P‐value	Variable P‐value
Age at neuter (months)	<6	1.82	1.15 to 2.88	0.011	0.017
	6–<12	Base			
	12–<24	0.88	0.58 to 1.34	0.548	
	≥24	0.78	0.44 to 1.40	0.406	
Time‐dependent effect Age at neuter (months)	<6 X Time interaction	0.75	0.63 to 0.90	0.002	0.001
	6–<12 X Time interaction	Base			
	12–<24 X Time interaction	1.10	0.95 to 1.28	0.188	
	≥ 24 X Time interaction	1.23	0.94 to 1.60	0.129	
Bodyweight (kg)	<10.0	Base			<0.001
	10.0 to <20.0	2.05	1.46 to 2.89	<0.001	
	20.0 to <30.0	3.54	2.49 to 5.03	<0.001	
	≥ 30.0	3.86	2.66 to 5.60	<0.001	
	Not recorded	7.46	3.69 to 15.11	<0.001	
Breed	Crossbreed – non‐designer	Base			<0.001
	Irish setter	15.45	6.19 to 38.54	<0.001	
	Dalmatian	4.33	2.28 to 8.25	<0.001	
	Hungarian vizsla	3.52	1.41 to 8.77	0.007	
	Dobermann	3.24	1.29 to 8.15	0.013	
	Weimaraner	3.07	1.40 to 6.75	0.005	
	Rottweiler	2.73	1.37 to 5.44	0.004	
	English springer spaniel	2.23	1.40 to 3.56	0.001	
	West Highland white terrier	2.21	1.16 to 4.23	0.016	
	Boxer	1.71	0.94 to 3.12	0.079	
	German shepherd dog	1.44	0.86 to 2.43	0.166	
	Siberian husky	1.37	0.59 to 3.16	0.466	
	Jack Russell terrier	0.93	0.48 to 1.79	0.825	
	Border collie	1.15	0.67 to 1.99	0.612	
	Purebreed – other	1.02	0.75 to 1.38	0.915	
	Labrador retriever	0.98	0.67 to 1.43	0.907	
	Crossbreed – designer	0.80	0.45 to 1.43	0.456	
	Staffordshire bull terrier	0.80	0.48 to 1.33	0.395	
	Cocker spaniel	0.55	0.28 to 1.09	0.087	
Vet group	1	Base			0.027

Age at neuter interacted with time

CI confidence interval

Compared to bitches neutered at 6 to <12 months, bitches that were neutered at <6 months had significantly increased hazard of early‐onset UI diagnosis within the first year following neuter (HR 1.82; 95% CI 1.15 to 2.88; P=0.011), but this increased hazard reduced 0.75‐fold (95% CI 0.63 to 0.90; P=0.002) for each subsequent year following neuter (*e.g*. HR year 2 = 1.82 × 0.75 = 1.37, HR year 3 = 1.82 × 0.75 × 0.75 = 1.02). However, there was no significant difference in the hazard of early‐onset UI diagnosis between bitches neutered at ≥12 months and those neutered at 6 to <12 months at any time point.

The final model identified eight breeds with increased hazard of early‐onset UI from the date of neuter compared to non‐designer crossbreds: Irish setter (HR 15.45; 95% CI 6.19 to 38.54; P<0.001), Dalmatian (HR 4.33; 95% CI 2.28 to 8.25; P<0.001), Hungarian vizsla (HR 3.52; 95% CI 1.41 to 8.77; P=0.007), Dobermann (HR 3.24; 95% CI 1.29 to 8.15; P=0.013), Weimaraner (HR 3.07; 95% CI 1.40 to 6.75; P=0.005), Rottweiler (HR 2.73; 95% CI 1.37 to 5.44; P=0.004), English springer spaniel (HR 2.23; 95% CI 1.40 to 3.56; P=0.001) and West Highland white terrier (HR 2.21; 95% CI 1.16 to 4.23; P=0.016). There were no breeds with significantly reduced hazard compared with non‐designer crossbreds. Increasing bodyweight (kg) was associated with an increasing hazard of early‐onset UI diagnosis from the date of neuter; bitches weighing ≥30.0 kg at time of neutering had 3.86 times the hazard (95% CI 2.66 to 5.60; P<0.001) compared with bitches weighing <10.0 kg. In addition, vet group was identified as a statistically significant variable (P=0.027) (Table 4).

## DISCUSSION

This cohort study of early‐onset UI (≤8 years) in bitches under primary veterinary care in the UK included an overall study population of 77,138 bitches and identified 493 incident cases. Incidence risk during the overall study period was 0.68%, while incidence rate increased with each category of increasing age. Neutered bitches showed increased hazard of early‐onset UI from date of birth compared with entire bitches. Neutering before 6 months of age was associated with significantly increased hazard of early‐onset UI diagnosis within the first year following neuter compared to neutering at 6 to <12 months. However, no significant difference in hazard of early‐onset UI from date of neuter was detected between neutering at ≥12 months compared to neutering at 6 to <12 months. Increased hazard of early‐onset UI diagnosis, from both date of birth in all bitches and date of neuter in the neutered subset of bitches, was also shown for heavier bitches and in particular breeds (namely the Irish setter, Dalmatian, Hungarian vizsla, Dobermann, Weimaraner and English springer spaniel).

UI in dogs is a condition that can be distressing for the owner, resulting in feelings of anger, disappointment and frustration in 10 to 20% of affected households (de Bleser *et al*. 2011). The direct welfare impact on the bitch includes increased risk of urinary tract infection and urine scald (Diesel *et al*. 2010). Increased risk of UI in bitches has been attributed to neutering (Holt & Thrusfield 1993). However, a recent systematic review reported that the evidence for an association between neutering and UI was weak and that further work was required to better understand any associations (Beauvais *et al*. 2012). Therefore, due to the welfare implications, the potential impact on the owner‐animal bond and the importance of the condition in the neutering decision‐making process, this cohort study was undertaken to further evaluate the condition and the role neutering plays in the development of early‐onset UI. Early‐onset UI was explored, since older or geriatric dogs may have other concurrent underlying conditions resulting in micturition disorders (Forsee *et al*. 2013) and age‐related degenerative changes that may be less influenced by neuter status and the choices that owners and veterinary surgeons make in early life. Studies including all UI cases may represent a variety of causes for UI, including age degeneration, and so studying early‐onset UI allows for more specific phenotype selection.

The incidence risk of early‐onset UI over the study period (January 1, 2010 to March 31, 2018) was calculated as 0.68%. However, the incidence rate of early‐onset UI increased from 0.0018 per dog year at risk in bitches aged 0 to <2 years to 0.0054 per dog year at risk in bitches aged 6 to 8 years, highlighting that the risk of early‐onset UI diagnosis varied over time. This is difficult to compare to earlier incidence estimates, as differing case definitions, study populations and study periods have been used in previous publications (Okkens *et al*. 1997, Thrusfield *et al*. 1998, Stocklin‐Gautschi *et al*. 2001, Angioletti *et al*. 2004). Nonetheless, previous work suggested a 5‐year incidence of 2.72% and estimated incidence rates in neutered and entire animals of 0.0174 and 0.0022 per animal‐year, respectively (Thrusfield *et al*. 1998). It is possible that the current study underestimated incidence risk and incidence rate, as bitches within our study population could have developed early‐onset UI, but may not have presented to a veterinarian and thus would not have been identified as cases. However, given the impact on the owner‐animal bond, it was considered likely that the owners of most bitches that develop incontinence were likely to seek veterinary assistance.

Neutering was identified as a major hazard associated with early‐onset UI in the multivariable analysis, which is consistent with previous findings that have reported neutering as a risk factor for UI (Thrusfield 1985, Holt & Thrusfield 1993, Thrusfield *et al*. 1998, O'Neill *et al*. 2017). In the current study, neutered bitches had 2.12 times the hazard of UI diagnosis within the first year after birth compared to those that were entire. The hazard increased by a factor of 1.23 for every subsequent year. A relative risk of 7.8 for neutered *versus* entire bitches was reported in a previous cohort study (Thrusfield *et al*. 1998). Relative risk is cumulative over an entire study, while HRs represent instantaneous risk over the study time period (Pfeiffer 2010). However, the hazard of early‐onset UI diagnosis in neutered bitches in the current study increased over time (with increasing age), and reached HRs of approximately 4.0 by 4 years of age. The present study included both congenital and acquired cases of UI, while the previous study focused on acquired UI cases only. Bitches with congenital UI tend to present as juveniles and thus are more likely to be entire at UI diagnosis, while bitches with acquired UI are usually adults and more likely to be neutered at UI diagnosis (Holt & Thrusfield 1993). This may partially explain why the hazard was lower initially, but subsequently increased.

Median time between neuter and first diagnosis of UI was 1.9 years, which is slightly lower than previous reports ranging from 2.3 to 5.0 years (Holt 1985, Stocklin‐Gautschi *et al*. 2001, de Bleser *et al*. 2011, Forsee *et al*. 2013, Byron *et al*. 2017). The difference in time between neuter and UI diagnosis in the current study compared to previous reports may be a result of different study subjects, different UI case definitions (because cases were not classified as congenital or acquired in the current study) and varying amounts of follow‐up time.

The proportion of neutered dogs (both male and female) in the UK has been estimated at 41.1% in a previous study using VetCompass data (O'Neill *et al*. 2014). The proportion of neutered bitches in the current study was slightly lower (35.9%), perhaps because the population was younger. However, the cohort study design allowed the time‐varying nature of neuter status to be taken in to account. Therefore, the current estimate may be more accurate than previous reports that have treated neuter status as a time‐independent variable.

Bitches neutered before 6 months had 1.82 times the hazard of early‐onset UI diagnosis within the first year following neuter compared to those neutered at 6 to <12 months. However, the HR decreased 0.75‐fold for every subsequent year, meaning that the increased hazard in bitches neutered at <6 months was only observed during the first 2 years following neuter (e.g. HR year 2 = 1.37, HR year 3 = 1.02). There was reduced information beyond year 3 (Fig 2), therefore evidence for any subsequent protective effect is limited. The accelerated time to early‐onset UI in early‐neutered bitches suggests a hormonal aetiology with any effect of neutering observed relatively soon after the procedure, whereas it may be that other more important factors such as anatomy and concurrent disease play a pivotal role in later UI development. There was no significant increase or decrease in the hazard of early‐onset UI diagnosis for bitches neutered at 12 to <24 or ≥24 months compared to those neutered at 6 to <12 months and no significant time‐varying effect. Nonetheless, the point estimates do suggest a 12% reduction in early‐onset UI diagnosis (within the first year following neuter) for bitches aged 12 to <24 months and a 22% reduction in early‐onset UI diagnosis for bitches aged ≥24 months compared with those aged 6 to <12 months. However, these results must be interpreted cautiously due to the substantial imprecision and wide CIs, limiting the evidence for an effect.

There is conflicting evidence reported on whether there is an association between age at neuter and UI diagnosis (Thrusfield *et al*. 1998, Spain *et al*. 2004, de Bleser *et al*. 2011, Beauvais *et al*. 2012, Hart *et al*. 2016). A US study reported a greater incidence of UI in bitches that were neutered before 3 months of age (Spain *et al*. 2004). In the current study, no cases and only five non‐cases were neutered before 3 months, suggesting that the increased hazard of early‐onset UI diagnosis in bitches aged *<*6 months identified in our study was referable primarily to bitches aged 3 to <6 months. It is likely that the bitches neutered before 6 months had not yet had their first oestrus, suggesting that associations between early neutering and early‐onset UI may be linked with oestrus. A recent US study reported that bitches weighing >25 kg neutered within their first year of life had a decreased hazard of UI for every 1‐month delay in neutering supporting an early age association (Byron *et al*. 2017).

Many of the breeds predisposed to early‐onset UI in the current study have also been previously reported at‐risk of UI: Irish setter, Dalmatian, Weimaraner, Dobermann, boxer, English springer spaniel and Rottweiler (Holt & Thrusfield 1993, Arnold 1997, O'Neill *et al*. 2017). As these breeds have been recognised as being at‐risk of UI in previous literature, it is possible a diagnosis was reached more readily in these cases. However, given a diagnosis of UI is made relatively easily, it is likely that where dogs of other breeds presented with appropriate clinical signs they too would have a diagnosis of UI made. In addition, however, the current study also identified novel predispositions in the Hungarian vizsla, shar‐pei and West Highland white terrier which have not been previously reported. This may be because previous smaller studies had less power to evaluate the associations for less common breeds, may indicate a changing risk or demographics for breeds over time or may be a result of differing UI case definitions. It should be noted that breeds with a minimum of six UI cases were included in the current study. Although the overall dataset was large, the individual breed numbers were relatively small for some breeds. Therefore, the P‐values should be interpreted alongside the CIs.

The West Highland white terrier was the only smaller breed dog at increased hazard of early‐onset UI from date of neuter. This breed is predisposed to atopic dermatitis, with up to 25% of the breed affected (DeBoer & Hill 1999). Atopic dermatitis is often treated with glucocorticoids, which frequently results in polyuria and polydipsia (Olivry *et al*. 2010), therefore this effect may promote UI in the West Highland white terrier. The shih‐tzu, Staffordshire bull terrier and cocker spaniel were at reduced hazard of early‐onset UI diagnosis from date of birth, consistent with previous work (O'Neill *et al*. 2017).

Increasing bodyweight (kg) was associated with an increased hazard of early‐onset UI diagnosis from both date of birth and date of neuter. These results are comparable to previous studies that also identified increased UI in heavier bitches (Okkens *et al*. 1997, Stocklin‐Gautschi *et al*. 2001, Angioletti *et al*. 2004, de Bleser *et al*. 2011, Forsee *et al*. 2013, O'Neill *et al*. 2017). A recent study highlighted that breed and bodyweight are highly correlated, therefore identifying which phenotypic characteristic represents the major association can be challenging (O'Neill *et al*. 2017). The current study reported weight as a risk factor for receiving a diagnosis, which includes age effects, as opposed to a risk factor for having a diagnosis. However, it is possible that including non‐adult bodyweights may have overestimated larger bodyweights as a risk factor for early‐onset UI diagnosis, although the results are in line with previous reports (O'Neill *et al*. 2017). Additionally, the bodyweight of controls was recorded as that closest to the date of final EPR, which might have overestimated bodyweight of controls compared to cases as they would have been slightly older at the time of weight measurement. This date was selected to provide a consistent date given there was not necessarily a comparable “date of diagnosis” for all controls.

Though this study represents the largest cohort study undertaken to date from practice attending data, there are limitations to the current study. The median follow‐up time from date of birth of non‐cases was relatively short (1.5 years). It may be that bitches censored before 1.5 years did not have a reason to visit a veterinarian since their last visit, but would do so if a health concern such as UI arose. However, not all bitches with UI may present to a veterinarian or bitches may develop UI but no longer be registered at the participating practices, which would impact on the follow‐up time in this study. In addition, there are bitches that may subsequently develop UI beyond the end of the study period.

We used the term “early‐onset UI” in the study, which could imply juveniles, but this term was used to encompass dogs diagnosed at any age up to 8 years. We did not attempt to classify cases as congenital and acquired because the underlying pathogenesis in most cases was unknown and may involve complex interactions between many different aetiological factors. However, in the bitches diagnosed at a young age, it is possible that there was an underlying delayed‐onset congenital component, thus a different pathophysiology to UI in adult bitches. The neuter status of cases was taken at the date of early‐onset UI diagnosis, thus in all neutered cases it is likely that neutering had an effect on the subsequent development of UI, despite the precise underlying cause being unknown. Attempts were made to extract data on the stated cause of UI and duration of UI before diagnosis but this information was often non‐specific and was inconsistently reported in the EPR and therefore was not deemed sufficiently reliable to include in the analysis. The current study did not evaluate the surgical method of neuter or the timing of neutering in relation to first oestrus due to limited availability of this information from the clinical records.

Using stepwise selection to build a multivariable model has its disadvantages, affecting both interpretation and prediction. However, a manual backward elimination stepwise approach was adopted using the variable likelihood ratio test p values as the elimination criteria to minimise limitations of model development (Dohoo 2010). Although not a primary risk factor, vet group was included as a fixed effect to account for confounding and variation between groups, although this affects generalisability of findings and might artificially deflate standard error estimates and, hence, precision.

## CONCLUSION

Neutering itself and age at neuter were identified as important risk factors associated with early‐onset UI. Neutering was associated with an increased hazard of early‐onset UI from date of birth. Bitches neutered before 6 months of age had increased hazard of early‐onset UI diagnosis within the first 2 years following neutering. The decision to neuter a bitch is based on many factors, not just UI risk alone. However, the contribution to decision‐making driven by UI may need to be greater for the high‐risk breeds and bitches with larger bodyweights. The results of this study suggest that early‐age neuter (<6 months) should be carefully considered, particularly in high‐risk breeds and bitches with larger bodyweights (or larger projected bodyweights), unless there are major other reasons for performing it.

### Conflict of interest

The authors have no conflicts of interest to declare.

## Data Availability

The datasets generated and analysed during the current study are not publicly available due to their use in ongoing primary research but may be made available from the corresponding author on reasonable request.
